# An Unusual Presentation of Respiratory Dysfunction in Parkinson’s Disease: A Case Study

**DOI:** 10.7759/cureus.77101

**Published:** 2025-01-07

**Authors:** Lily D Rundquist, Sarah E Lyons, Rosa J Moljo, Cyril Blavo

**Affiliations:** 1 Medicine, Nova Southeastern University Dr. Kiran C. Patel College of Osteopathic Medicine, Davie, USA; 2 Family Medicine, Nova Southeastern University Dr. Kiran C. Patel College of Osteopathic Medicine, Clearwater, USA; 3 Public Health and Pediatrics, Nova Southeastern University Dr. Kiran C. Patel College of Osteopathic Medicine, Clearwater, USA

**Keywords:** exhalation, parkinson' s disease, respiratory center, respiratory dysfunction, respiratory rigidity

## Abstract

Parkinson’s disease (PD) is a progressive neurodegenerative disorder affecting around 10 million people worldwide. It is primarily associated with the loss of dopaminergic neurons in the substantia nigra pars compacta, though the exact mechanism is unclear. PD is characterized by resting tremor, muscular rigidity, bradykinesia, and postural instability. These motor symptoms, resulting from impaired muscle function, can lead to dysphagia and respiratory dysfunction. It is suggested that the muscular rigidity and bradykinesia in PD may impair repetitive motor actions, negatively impacting respiration. While less studied, PD-related respiratory dysfunction could stem from obstructive, restrictive, or CNS disturbances. This case report discusses a 69-year-old male with PD and dementia presented at an assisted living facility in Palmar Sur, Costa Rica, with difficulty exhaling after deep inhalation. Physical examination revealed typical PD symptoms (resting tremor, muscular rigidity, bradykinesia, postural instability) along with hypotension, bradycardia, and bradypnea. Respiratory assessment showed he could inhale but had difficulty exhaling, suggesting a myotonic-like pathology. The cogwheel-like rigidity during exhalation is unique and points to a myotonic-like respiratory dysfunction related to his PD, a presentation not commonly observed in PD patients. Research suggests respiratory impairments in PD may result from issues with central ventilatory control, restrictive lung disease (reduced lung capacity), or obstructive lung disease (blocked airflow). Restrictive mechanisms might stem from thoracic rigidity and posture changes, while obstructive factors could be exacerbated by the patient’s smoking history. Respiratory muscle fatigue may arise from the demands of repetitive ventilation. Additionally, degeneration in the brain’s respiratory center, possibly linked to α-synuclein deposits, could affect respiratory regulation. Osteopathic Manipulation Treatment (OMT) may help alleviate these symptoms by improving thoracic flexibility and respiratory function. Overall, this case highlights the varied presentations of PD, focusing on the lens respiratory dysfunction, possibly due to respiratory muscle rigidity or central nervous system involvement. Recognizing these symptoms is crucial for early intervention and preventing complications like aspiration pneumonia, a major cause of morbidity and mortality in PD. Further research is needed to better understand the relationship between PD and respiratory dysfunction.

## Introduction

Parkinson’s disease (PD) is a progressive neurodegenerative disorder affecting an estimated 10 million people worldwide [1]. It is proposed that the muscle rigidity and bradykinesia seen in PD impair repetitive motor actions, which could be contributing factors in the respiratory dysfunction observed in many PD patients. This dysfunction could also be attributed to other factors such as decreased lung capacity, thoracic kyphosis, and/or respiratory muscle fatigue. Though the mainstay treatment for PD is dopamine agonists, such as Levodopa, there is no current medication to halt the progression of the disease [2]. In the United States, the incidence of PD is predicted to grow from 1 million to 1.2 million cases by 2030. This trend in PD is predicted to increase rapidly [3]. There is a need for the scientific community to better understand the pathophysiology of this progressive disease, particularly how and why the respiratory system is affected. As it is currently understood, the pathology of PD is rooted in the loss of dopaminergic neurons in the substantia nigra pars compacta (SNpc), but the pathogenesis behind this is not completely understood [4]. One proposed mechanism of this neuron loss is the formation and inclusion of α-synuclein-containing Lewy bodies, resulting in gradual neuronal death [5]. The neuronal death results in a significant decline in SNpc dopaminergic neurons, leading to the classic motor deficits seen in PD patients [6]. Clinically, PD is characterized by resting tremor, muscle rigidity, bradykinesia, and postural instability [7]. Impairment of muscle function has been known to cause dysphagia as well as respiratory dysfunctions. Here we report a case in which a patient diagnosed with PD presented with dysfunctional exhalation during respiratory cycles and discuss proposed mechanisms for this dysfunction.

## Case presentation

A chart review was performed, and appropriate consent was obtained for a 69-year-old male with a medical history of PD and dementia who presented with difficulty exhaling, specifically following deep inhalation. The patient was a non-verbal, non-mobile, long-term resident of an assisted living facility in Palmar Sur, Costa Rica. The exact onset of his symptoms is unknown as it was not mentioned in his medical records. He was formally diagnosed with PD in 2006 and has been taking the following medications: Acetaminophen 500 mg and Aspirin 100 mg in the morning, Levodopa 125 mg three times a day, Biperiden 1 mg in the morning and 2 mg in the afternoon, Clonazepam 2 mg in the evening, Risperidone 0.5 mg in the evening, and Famotidine 40 mg in the evening. Biperiden (Akineton) is an anticholinergic agent that reduces oral secretions commonly used with Levodopa to treat PD; Biperiden is currently prescribed in Costa Rica but was discontinued in the United States due to potential liver toxicity [8]. The assisted living facility director reported that the patient smoked an unknown amount of tobacco for an unknown duration of time. His family reported that he smoked marijuana and drank a significant amount of alcohol throughout his life. There is no report of exposure to potential PD-associated chemicals, such as carbamates or other pesticides. His medical record revealed a history of psychiatric and social problems, as well as dementia. He has a history of bouts of agitation and irritability, as well as aggressive interactions with residents and staff. Due to his non-verbal state, further subjective history could not be obtained. The patient’s past medical history is significant for myocardial infarction at age 64.

On physical exam, the patient displayed resting tremors, muscle rigidity, bradykinesia, and postural instability. He also exhibited dysphagia on direct observation of eating, a lip quiver, and a pill-rolling tremor of his hands. His vital signs were significant for hypotension, bradycardia, and bradypnea. Additionally, the patient exhibited severe cognitive deficits and was unable to speak. The patient was oriented to person, but not to place, or time and exhibited limited alertness. He was able to follow simple instructions at peak alertness. Of note, the patient also exhibited signs of age regression, such as a positive grasp reflex and frequent oral exploration.

During his respiratory assessment, the patient was able to inhale on command but had difficulty with exhalation. Due to his challenges with verbal communication, visual cues were used to conduct the physical examination. It was necessary to prompt him to begin taking deep breaths. After he began to intentionally take deep inhalations, his difficulty with exhalations became noticeable. At first, it was believed that there was a miscommunication and that the patient was holding his breath, but after observing several respiratory cycles it was concluded that deep inhalation resulted in delayed and rigid exhalation. The patient appeared to have trouble expelling the air on exhalation. The patient’s current medications could interact and contribute to the observed respiratory symptoms and impaired cognition. Biperiden, in particular, may exacerbate cognitive issues in elderly patients and could potentially affect respiratory muscle function [9]. Additionally, Clonazepam and Risperidone can have sedative effects that impair respiratory drive. Given his current symptoms, reconsideration of the medication regimen is warranted. Reducing doses or switching to alternative medications with fewer side effects could have helped manage his PD, dementia, and respiratory dysfunction more effectively.

An MRI of this brain to assess for neurodegenerative changes, and pulmonary function tests (PFTs) could have provided valuable insights on the patient’s condition. Unfortunately, MRI and PFTs were not available diagnostic tools at the time of the exam. The patient also would not have been unable to cooperate during PTFs to formally assess his pulmonary function. Following this assessment, a comprehensive management plan was developed. This included adjustments to his medication regimen, respiratory therapy, and ongoing monitoring of his pulmonary function. However, due to the limited resources and capabilities of the facility, only minimal changes were able to be made in order to implement this plan. Given the complexity of this case, a multidisciplinary team approach involving neurology, pulmonology, geriatrics, and psychiatry could have enhanced the quality of care for this patient. Regular consultations with these specialists could have helped optimize treatment strategies and improve the patient's overall well-being. Formal monitoring of the patient’s respiratory condition and PD symptoms were recommended to his primary care team. Regular physician follow-up would allow for timely adjustments to the treatment plan based on the patient’s evolving needs and responses to therapy. The patient was non-verbal and had dementia, making informed consent challenging. Consent for treatment and chart review was obtained from his family, who were acting on the patient’s behalf.

## Discussion

Typical idiopathic PD is the most common form of Parkinson’s disease [10]. The symptoms include skeletomuscular effects such as resting tremors, muscle rigidity, and bradykinesia, as well as non-motor symptoms such as constipation, orthostatic hypotension, depression, dysphagia, and dysarthria [11]. Discussion with the assisted living facility revealed that since his diagnosis in 2006, the patient has progressed through the stages of PD, experiencing increasing motor symptoms and notable cognitive decline. He is currently in the fifth stage due to being either bedridden or confined to a wheelchair, as well as his need for constant care and assistance. The patient’s history of resting tremors, lip quivers, pill-rolling tremors, and postural instability, as well as long-term treatment with Levodopa are consistent with the diagnosis of idiopathic PD. There were no documented respiratory symptoms prior to this assessment, suggesting that the current respiratory dysfunction may be a new development. The social history indicates that the patient smoked tobacco and marijuana; however, the duration and frequency of these habits remain unclear, making it challenging to fully understand their potential impact on his respiratory condition. Pinpointing the exact onset of this patient’s respiratory symptoms was difficult due to the inability of the patient to verbally initiate his complaints with us as well as his primary physician. It’s also notable that the onset of his respiratory difficulty occurs when provoked to deeply inhale, therefore the patient does not present with any obvious wheezing or signs of respiratory distress that would prompt initial investigation.

The patient’s inability to relax upon exhalation during his normal respiration is an unusual presentation of this complex disease. The delayed exhalation noted in this patient could be due to the skeletal muscle abnormalities seen in PD. The respiratory dysfunction appeared to be related to PD, as there is evidence of a link between PD and impairment of repetitive motor movements [12]. Tzelepis et al. suggest that repetitive ventilation could contribute to respiratory muscle fatigue, similar to that seen in other muscle groups of PD patients [12]. It was unclear in this case whether the respiratory dysfunction observed in the patient was due to an impairment of the accessory muscles of respiration, the smooth muscles in the bronchi and bronchioles, or his respiratory center. A 2017 review by Torsney and Forsyth has highlighted the prevalence of respiratory dysfunction in PD and discussed potential underlying mechanisms such as neural pathway degeneration and muscle rigidity, reinforcing the need for further investigation in similar cases [13].

Respiratory dysfunctions seen in PD patients are not well understood and could be multifactorial. D’Arrigo et al. maintain that this respiratory dysfunction is due to an interplay of different mechanisms including central ventilatory control, restrictive disease, obstructive disease, and drug-related side effects [14]. Considering a differential diagnosis in this case is essential; conditions such as obstructive pulmonary disease (e.g., chronic obstructive pulmonary disease, asthma), neuromuscular disorders (e.g., amyotrophic lateral sclerosis, myasthenia gravis), or restrictive lung diseases (e.g., pulmonary fibrosis) may also contribute to the patient's difficulty in exhaling.

It is unclear whether these symptoms are attributed primarily to his PD diagnosis or his social history. The observed inability of the patient to properly initiate exhalation could be a combination of the two. Direct observation of the patient’s respirations revealed that the patient was able to take a deep breath, but that he was unable to relax to exhale and expel the air. Alternative respiratory assessment tools that were unavailable to us, such as capnography or pulse oximetry, might have provided additional insights into his respiratory function without relying heavily on his cooperation. Current research suggests an association between PD and progressive respiratory dysfunction [15]. Kaminsky et al. observed significant respiratory decline in PD patients compared to healthy individuals of a similar age and this article proposes that respiratory dysfunction in PD is attributed to a decrease in lung volume and progressive respiratory muscle weakness [15]. Other articles suggest an association between PD and a restrictive pattern of lung disease [16]. This is predominantly noted in cases where patients exhibit significant thoracic kyphosis. Santos et al. attribute respiratory symptoms in PD patients to postural changes, general rigidity, and the sedentary lifestyle seen in later stages of PD. They suggest that the lack of exercise and voluntary mobility causes muscle atrophy and muscle weakness, impacting pulmonary function [16].

**Figure 1:**
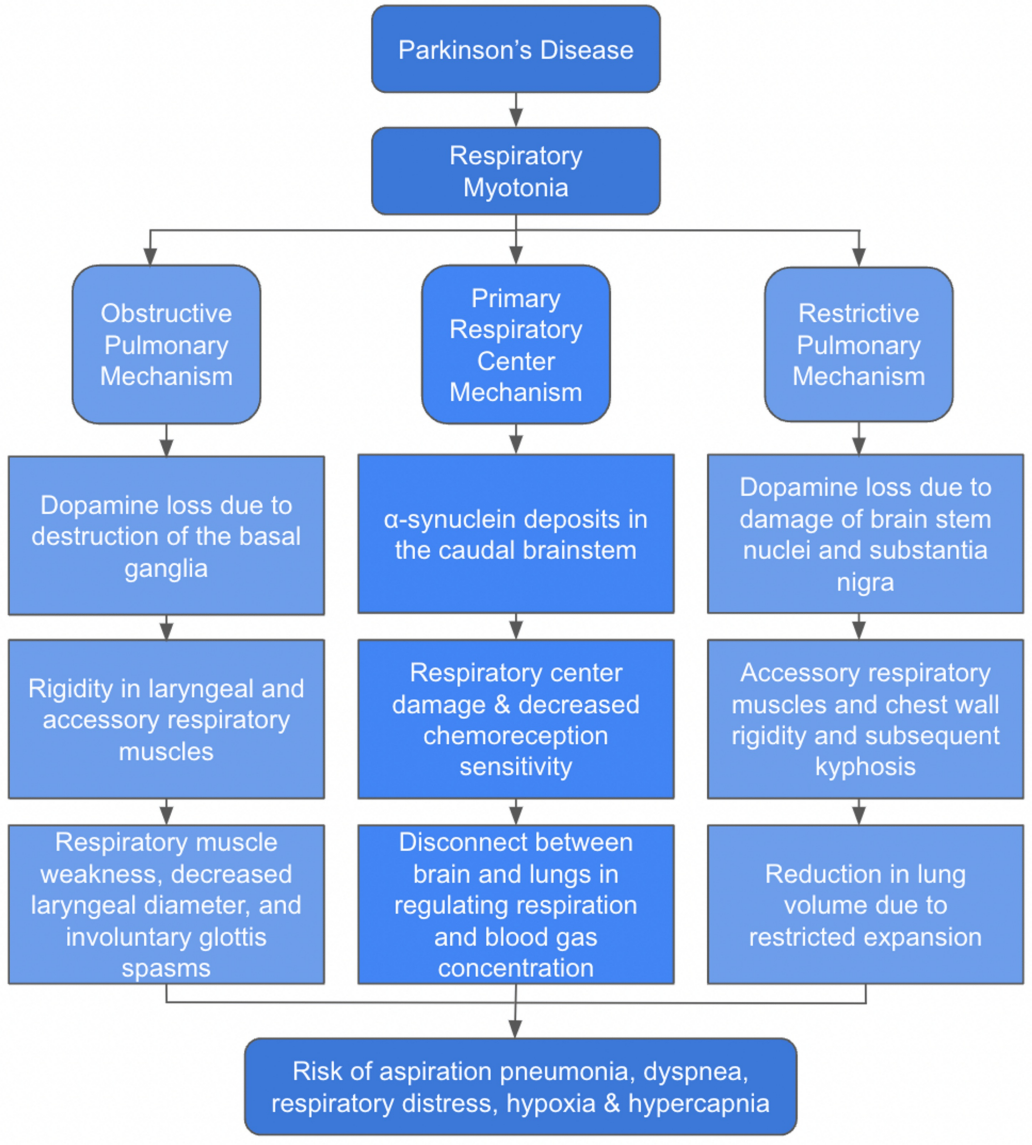
Depiction of potential mechanisms for respiratory impairment in PD

Degeneration of function in the respiratory center of the brain is another theory on the pathophysiology behind respiratory difficulty in PD patients (Figure 1) [17]. Addressing thoracic kyphosis and postural instability of PD patients could have a positive impact on respiratory function, particularly in those with restrictive lung disease secondary to thoracic kyphosis. α-synuclein deposition in the caudal brainstem is postulated to impair the ability to initiate respiration. These deposits also damage the body’s ability to detect changes in chemoreception, causing the respiratory cycle to be less sensitive in response to changes in oxygen and carbon dioxide levels [18]. By compromising the body’s ability to detect blood gas concentrations, respiratory regulation is diminished. This aberration could potentially lead to hypoxemia and/or hypercapnia. The dysregulation between the brain and body could explain the dyspnea and respiratory myotonia in PD patients. Osteopathic Manipulation Treatment (OMT) is a treatment modality that has been shown to have success with PD patients, making it a viable treatment option to address the respiratory symptoms. OMT may also improve the quality of life in PD patients [19]. OMT has been shown to decrease respiratory rigidity and improve patient respiration through techniques such as thoracic diaphragm release, treatment of thoracic and rib somatic dysfunctions, and other methods of decreasing hypertonicity of primary and secondary respiratory muscles [20]. By focusing on these specific areas, respiratory efficiency can be significantly improved among PD patients who suffer from pulmonary dysfunction.

## Conclusions

This case study is significant because varying presentations in patients with PD help the scientific community develop a better understanding of the pathophysiology of the disease. This case highlights a patient with PD presenting with concomitant respiratory dysfunction of unclear etiology. Investigation of the underlying etiology revealed key findings about the potential origins of the dysfunction. Proposed mechanisms include respiratory muscle weakness, respiratory center degeneration, and restrictions in lung volume. Increased awareness of respiratory symptoms, such as those observed in this patient, could lead to earlier intervention and prevention of complications such as aspiration pneumonia, a major cause of mortality and morbidity in PD patients. Further research is needed to substantiate the relationship between PD and respiratory dysfunction.
